# Inflammatory Indexes as Predictive Biomarkers of Postoperative Complications in Oncological Thoracic Surgery

**DOI:** 10.3390/curroncol29050276

**Published:** 2022-05-08

**Authors:** Giorgio Carlo Ginesu, Panagiotis Paliogiannis, Claudio F. Feo, Maria Laura Cossu, Antonio Mario Scanu, Alessandro Fancellu, Alessandro Giuseppe Fois, Angelo Zinellu, Teresa Perra, Simone Veneroni, Alberto Porcu

**Affiliations:** 1 Department of Medical, Surgical and Experimental Sciences, University of Sassari, 07100 Sassari, Italy; ginesugc@uniss.it (G.C.G.); cffeo@uniss.it (C.F.F.); mlcossu@uniss.it (M.L.C.); scanu@uniss.it (A.M.S.); afancel@uniss.it (A.F.); agfois@uniss.it (A.G.F.); simone.veneroni@gmail.com (S.V.); alberto@uniss.it (A.P.); 2 Department of Biomedical Sciences, University of Sassari, 07100 Sassari, Italy; panospaliogiannis@gmail.com (P.P.); azinellu@uniss.it (A.Z.)

**Keywords:** thoracic surgery, lung cancer, biomarkers, inflammatory indexes, postoperative complications

## Abstract

The role of inflammatory responses in predicting outcomes in oncological thoracic surgery is still unclear. The aim of this study was to evaluate a series of blood count inflammation indexes as predicting factors for postoperative complications. We retrospectively studied 249 patients undergoing elective thoracic surgery in our institution between 2008 and 2020. A total of 184 patients underwent open surgery, and 65 underwent VATS. The neutrophil-to-lymphocyte (NLR), monocyte-to-lymphocyte (MLR), and platelet-to-lymphocyte (PLR) ratios, Systemic Inflammation Response Index (SIRI) were calculated preoperatively and on the first and fourth postoperative days, as well as a new derivative index, the Aggregate Inflammation Systemic Index (AISI). Univariate correlations evidenced a statistically significant association between the NLR at the fourth postoperative day and the occurrence of surgical complications in the global cohort (rho = 0.15, *p* = 0.03). A similar significant association with MLR on the fourth postoperative day is found in the open group (rho = −0.15, *p* = 0.048). NLR and LMR on the fourth postoperative day are associated with postoperative complications in the whole and open groups, respectively. Simple, easy-to-perform and inexpensive, blood cell count indexes may be useful in predicting complications in oncological thoracic surgery. A greater number of broader, prospective, randomized studies are necessary to confirm these findings.

## 1. Introduction

Lung cancer is estimated to be the second most diagnosed cancer worldwide, with an incidence of 2.2 million new cases/year, and the leading cause of cancer death, with 1.8 million deaths in 2020. It is the primary cause of cancer morbidity and mortality in men with a rate roughly two-times higher than in women, whose incidence, instead, ranks third after breast and colorectal cancer and second for mortality after breast cancer [1]. Even more frightening is the expectation of an increase in mortality in the future to come, reaching an incidence of 3 million deaths by the year 2035 [2].

The interplay between cancer and the immune and inflammatory response has been thoroughly described in the last twenty years [3,4]. In fact, tumour-promoting inflammation can be activated at several timings and by several stimuli, enhancing and favouring carcinogenesis and tumour growth, as well as metastatic processes [5].

Different indexes have been designed to reflect the inflammatory response in relation to ongoing oncological diseases. The neutrophil to lymphocyte ratio (NLR), the platelet to lymphocyte ratio (PLR), and the lymphocyte to monocyte ratio (LMR) are regarded as reliable markers for inflammatory processes. On this topic, these markers have been widely studied as prognostic oncological predictors of various cancers [6], along with lung cancers. Indeed, they were a significant independent prognostic factor for survival in patients diagnosed with diverse thoracic neoplastic processes [7,8,9,10,11].

Other combined analytical approaches, such as the Systemic Inflammation Response Index (SIRI) or the Aggregate Index of Systemic Inflammation (AISI), have been applied to predict the prognosis for benign and malignant diseases treated either with surgical or medical strategies [12,13,14,15,16].

Moreover, these tests are cheap and easy to be extrapolated from blood samples, making them optimal predictors in such contexts.

Thus, while their role as long-term survival prognostic factors is known, their capability to predict short-term postoperative complications in patients surgically treated for lung cancer is still uncertain.

Postoperative complications manifest in a wide proportion of patients after lung surgery (from 10% to 50%) [17]. This prolongs patients’ hospital stays and healthcare costs [18,19].

This study aims to present a retrospective analysis of such indicators in patients selected and treated with either open/thoracotomy or video-assisted thoracoscopic surgery (VATS) procedures at our department, evaluating their weight in predicting short and medium-term postoperative complications.

## 2. Materials and Methods

We made a retrospective study of 249 consecutive patients undergoing elective oncological thoracic surgery in our institution between 2008 and 2020. Included patients were diagnosed with primary thoracic tumors. According to the current literature, tumors were mostly adenocarcinomas (41%), followed by squamous cell carcinomas (34%) and small cell carcinomas (14%), and the remaining were lymphomas or other subtypes of primary tumors (11%). Metastatic cancers with pulmonary localizations were excluded. Patients undergoing emergency surgery and those with missing data were also excluded. Demographic, clinical, surgical, laboratory, and hospital stay information were retrieved from clinical and surgical records. Preoperatively, no major disease impacting the systemic inflammatory response was found in the cohort. Following preoperative assessment, patients were admitted to the ward the day before the scheduled operation. Two senior general surgeons with experience in thoracic procedures performed all operations. All patients signed an informed consent form for each procedure and for the use of their anonymous clinical data for research purposes; the study was conducted in accordance with the principles of the Declaration of Helsinki.

As part of the preoperative assessment and on the first and fourth postoperative days, complete blood counts were available in all patients. Fasting blood samples were obtained following standard procedures and protocols by current international and national guidelines and were analyzed in a certified laboratory. The neutrophil to lymphocyte (NLR), monocyte to lymphocyte (MLR), and platelet to lymphocyte (PLR) were calculated, as well as the Systemic Inflammation Response Index (SIRI), an index recently proposed by Qi et al. as a valuable prognostic marker in patients with pancreatic cancer after chemotherapy [12]. Furthermore, we studied a new derivative index, the Aggregate Inflammation Systemic Index (AISI), which included all the main inflammatory blood cell populations, including the platelets. The AISI was calculated by multiplying the number of neutrophils, monocytes and platelets and dividing the product by the number of lymphocytes [16].

The ability of the different indexes to predict postoperative complications was assessed using receiver operating characteristics (ROC) curve analysis and selection of optimal cut-off values for sensitivity and specificity according to the Youden index. The optimal cut-off for NLR, MLR and PLR was 3.1, 0.4 and 224, respectively. The optimal cut-off values for sensitivity and specificity in ROC analysis were 1.2 for SIRI and 221 for AISI. Although the sensitivity values were satisfactory, specificities were relatively low. Further investigations are required to confirm the observed predictive performance of these indexes.

Analyses were carried out in the whole cohort and in two separate patient groups: those who underwent open surgery and those who underwent VATS (Video-Assisted Thoracoscopic Surgery).

All results are expressed as mean values (mean ± SD) or median values (median and range). Variables distribution was assessed by the Shapiro–Wilk test. As appropriate, statistical differences between groups were compared using an unpaired Student’s *t*-test or Mann–Whitney rank-sum test. The differences between categorical variables were evaluated by a chi-squared test. Correlations between variables were assessed by Pearson’s correlation or Spearman’s correlation as appropriate. Statistical analyses were performed using MedCalc for Windows, version 15.4 64-bit (MedCalc Software, Ostend, Belgium).

## 3. Results

The main demographic, clinical and preoperative laboratory data are summarized in Table 1. Among the 249 patients enrolled, 184 were operated on with an open technique and 65 with a VATS procedure.

The median age in the whole cohort was 66 (IQR: 58–72) years, and no significant differences in the age of the patients were observed between the open and VATS group of patients. Approximately two-thirds of the patients were males, again with no significant difference between the groups. Current or former smokers were significantly more frequent in the open group. The duration of the surgical procedures, the hospital stay, and the incidence of complications were significantly higher in the open group; this can be explained considering the invasiveness and the types of the open procedures compared to the VATS procedures. Lobectomies were mostly done with an open approach, and no pneumonectomies were performed with VATS. Finally, some slight differences regarding the blood cell count, especially the derivative indexes, were detected between the open and VATS groups (Table 1).

Postoperative complications were present in 38 patients (22.4%). Of these, 38 patients with Clavien-Dindo (CD) grade II or inferior (15.2%), while 18 patients presented grade III or IV (7.2%); of this group, 3 patients (CD IIIa); 1 patient (CD IIIb); 14 patients (CD IVa, need for ICU after surgery).

**Figure 1:**
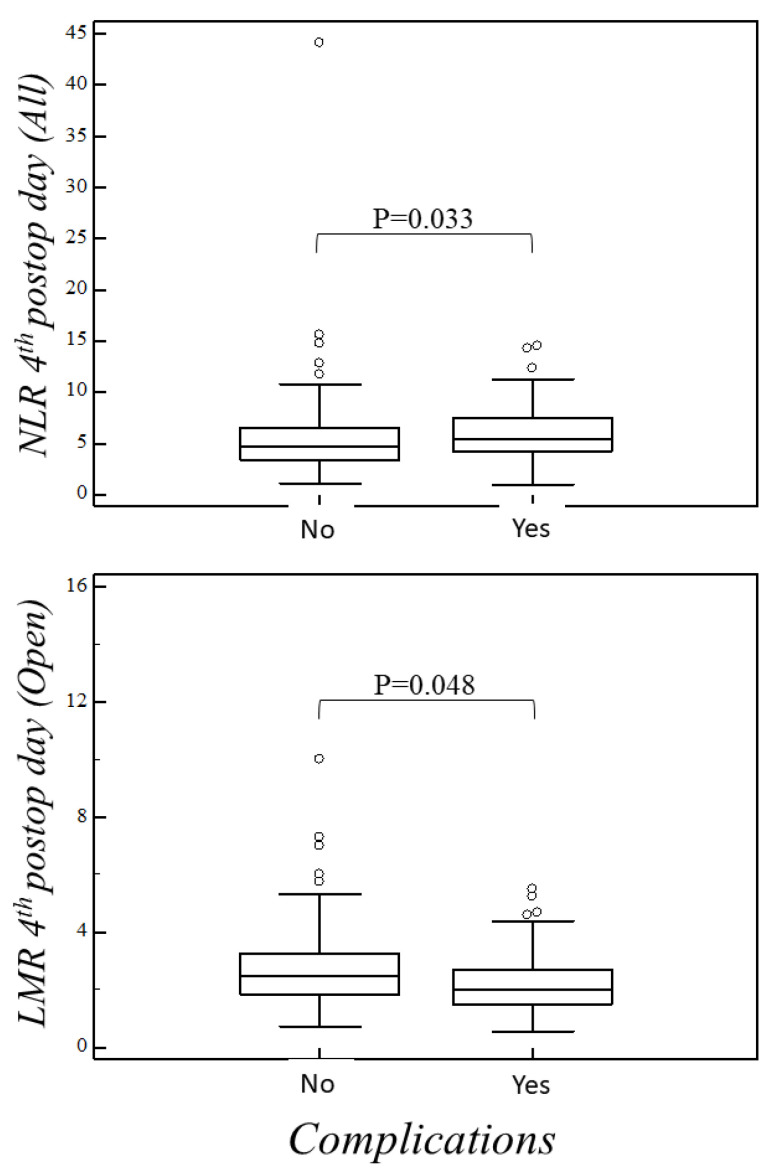
NLR fourth postop day (all patients). No complications vs. complications median 4.680 (IQR: 3.455–6.565) vs. 5.500 (IQR: 4.235–7.455), *p* = 0.033. LMR fourth postop day (open surgery). No complications vs. complications median 2.500 (IQR: 1.830–3.250) vs. 2.000 (IQR: 1.500–2.710), *p* = 0.048.

Univariate correlations between the inflammatory cell indexes and the incidence of complications evidenced a statistically significant association between the NLR at the fourth postoperative day and surgical complications in the global cohort (rho = 0.15, *p* = 0.03). This association was not confirmed in the open and VATS groups studied separately (Table 2). A similar significant association was found between MLR on the fourth postoperative day and surgical complications in the open group (rho = −0.15, *p* = 0.048). Figure 1 shows the fourth postoperative day NLR and MLR differences found in relation to the complications. In any case, no significant associations were detected between the basal preoperative or first postoperative day values of the indexes examined and the occurrence of postoperative complications.

Univariate correlations between the inflammatory cell indexes and hospital stay evidenced a statistically significant association in the global cohort and the open group, as shown in Table 3.

## 4. Discussion

In recent years, inflammatory response indexes, obtained from complete blood count, have been developed and studied as possible predictors both for benign and malignant pathologies, as it is known as the interplay of the immune system within the cancer environment.

These refer to neutrophil to lymphocyte ratio (NLR), platelet to lymphocyte ratio (PLR) and lymphocyte to monocyte ratio (LMR), as well as the systemic inflammation response index (SIRI) and the aggregate index of systemic inflammation (AISI).

Specifically, in the thoracic surgical field, they have been proposed to predict the prognosis in oncological patients. In the same way, they have also been analyzed for the preoperative prediction of hospital stay in elective thoracic open surgery [20]. Likewise, few studies have determined the relationship between such parameters with the risk for postoperative complications after major surgery [21,22,23,24,25,26].

The present study analyzes a sample of 249 patients, among which 56 patients (22.48%) showed a postoperative adverse event. These ranged from minor complications, not affecting the overall recovery to major complications. Common events were subcutaneous emphysema, alveolopleural fistula, blood transfusion and tachyarrhythmia, among others.

The aforementioned inflammatory response indexes analysis resulted in being statistically relevant for NLR and LMR on the fourth postoperative day in predicting postoperative adverse events for the whole cohort (both open and VATS techniques) and the open surgery group, respectively.

The statistical analysis did not prove the importance of these indexes in the preoperative and first postoperative day blood samples, which yielded negative results. This invariably reduced expectations of their predictive capacity. However, their relevance on the fourth postoperative day is clear and could be useful in managing those patients without still an evident clinical manifestation of any adverse event, thus directing particular attention to them.

In the same direction, a well-designed study by Lan et al. revealed the significance of preoperative PLR and NLR correlated to an increase in postoperative pulmonary complications in NSCLC patients treated with radical open lung resection [27].

Such divergences can be associated with the selection of patients, as the present study, even if with a larger sample size, intended to analyse the cohort of thoracic surgery patients as a whole, possibly showing differences among subgroups but also a greater propensity to biases.

Other study limitations were mainly the retrospective methodology and the size of the sample.

However, the results presented in this study, along with the currently available literature, give relevance to the possibility of implying such inflammatory indexes as predictors for postoperative complications even in the thoracic surgical field.

Laboratory implementation of these inflammatory indexes into the routine blood tests should be considered to better tail their evolution in the postoperative period along with the patient clinical response.

## 5. Conclusions

The present study evidenced that the NLR and LMR measured on the fourth postoperative day were associated with postoperative complications in the whole cohort and in patients who underwent open thoracic surgery, respectively. Our findings suggest that simple, easy-to-perform, and inexpensive blood cell count indexes may be useful in predicting complications in oncological thoracic surgery. Further and more extensive studies are necessary to confirm these findings.

## Figures and Tables

**Table 1:** Main demographic, clinical and basal laboratory features of the patients included in the study.

	Global Cohort (*n* = 249)	Open Surgery (*n* = 184)	VATS (*n* = 65)	*p*-Value
Age, years	66 (58–72)	66 (59–72)	64 (50–72)	0.21
Gender, females (%)	67 (26.9)	45 (24.5)	22 (33.8)	0.14
BMI	25.4 ± 4.1	25.4 ± 4.1	NA	NA
Non-smokers, (%)	48 (19.3)	27 (14.7)	21 (32.3)	0.002
Pack Years	37 (16–60)	38 (17–60)	31 (10–60)	0.5
ASA score	I 89; II 83;	I 73; II 61;	I 16; II 22;	0.085
	III 60; NA 17	III 38; NA 12	III 22; NA 5	<0.000001
Surgery duration, hours	3.0 (2.1–4.0)	3.5 (2.5–4.5)	2.0 (1.0–2.4)	<0.000001
Hospital Stay, days	8.0 (5.0–10.0)	9.0 (7.0–11.0)	5.0 (3.0–6.5)	0.036
Complications, (%)	58 (23.3)	49 (26.6)	9 (13.8)	0.21
Surgery Type				<0.0001
Lobectomy, n (%)	139 (55.8)	129 (70.1)	10 (15.4)	
Wedge resection, n (%)	67 (26.9)	31 (16.8)	36 (55.4)	
Pneumonectomy, n (%)	9 (3.6)	9 (4.9)	0 (0)	
Explorative procedure, n (%)	17 (6.8)	6 (3.2)	11 (16.9)	
Other, n (%)	17 (6.8)	9 (4.9)	8 (12.3)	
Blood Cell Count—Indexes				
Lymphocytes, (×109/L)	1.9 (1.5–2.4)	1.9 (1.5–2.4)	2.0 (1.5–2.5)	0.77
Monocytes, (×109/L)	0.5 (0.4–0.6)	0.5 (0.4–0.7)	0.4 (0.3–0.6)	0.05
Neutrophils, (×109/L)	4.6 (3.5–5.7)	4.5 (3.5–5.7)	4.7 (3.0–5.4)	0.28
Platelets, (×109/L)	255 (209–333)	256 (210–333)	248 (208–331)	0.80
RDW, (fL)	13.7 (12.9–14.8)	13.8 (13.0–14.8)	13.4 (12.7–14.8)	0.16
MPV, (fL)	8.3 (7.6–8.9)	8.3 (7.7–9.0)	8.1 (7.4–8.6)	0.42
NLR	2.3 (1.7–3.3)	2.4 (1.7–3.3)	2.0 (1.5–3.3)	0.17
PLR	137 (98–189)	137 (101–188)	139 (94–190)	0.79
LMR	4.4 (2.8–5.3)	3.8 (2.8–5.0)	4.5 (2.8–6.7)	0.03
SIRI	1.1 (0.7–1.8)	1.2 (0.8–1.8)	1.0 (0.5–1.7)	0.05
AISI	281 (163–565)	316 (181–573)	246 (121–448)	0.05

Data are presented as mean ± standard deviation or median (interquartile range). Abbreviations: VATS: video-assisted thoracic surgery; RDW, red cell distribution width; MPV, mean platelet volume; NLR, neutrophil to lymphocyte ratio; PLR, platelets to lymphocyte ratio; LMR, lymphocyte to monocytes ratio; SIRI, systemic inflammation response index; AISI, Aggregate inflammation systemic index; NA, not available.

**Table 2:** Blood cell count indexes and postoperative complications in the global cohort and the open and VATS surgery groups.

		Complications (All)	Complications (Open)	Complications (Vats)
**NLR**	Preoperative	rho = 0.00, *p* = 0.96	rho = 0.03, *p* = 0.65	rho = −0.18, *p* = 0.17
	1st postop day	rho = −0.07, *p* = 0.29	rho = −0.10, *p* = 0.19	rho = 0.03, *p* = 0.84
	4th postop day	rho = 0.15, *p* = 0.03	rho = 0.14, *p* = 0.07	rho = 0.14, *p* = 0.37
**PLR**	Preoperative	rho = 0.01, *p* = 0.87	rho = 0.01, *p* = 0.88	rho = 0.00, *p* = 0.92
	1st postop day	rho = −0.03, *p* = 0.65	rho = −0.04, *p* = 0.63	rho = 0.02, *p* = 0.90
	4th postop day	rho = 0.10, *p* = 0.17	rho = 0.09, *p* = 0.24	rho = 0.12, *p* = 0.44
**LMR**	Preoperative	rho = −0.03, *p* = 0.60	rho = −0.06, *p* = 0.39	rho = 0.13, *p* = 0.32
	1st postop day	rho = 0.02, *p* = 0.74	rho = 0.00, *p* = 0.96	rho = 0.15, *p* = 0.26
	4th postop day	rho = −0.11, *p* = 0.10	rho = −0.15, *p* = 0.048	rho = 0.07, *p* = 0.63
**SIRI**	Preoperative	rho = 0.27, *p* = 0.68	rho = 0.07, *p* = 0.38	rho = −0.18, *p* = 0.17
	1st postop day	rho = −0.05, *p* = 0.43	rho = −0.04, *p* = 0.61	rho = −0.17, *p* = 0.22
	4th postop day	rho = 0.11, *p* = 0.12	rho = 0.14, *p* = 0.07	rho = −0.07, *p* = 0.63
**AISI**	Preoperative	rho = 0.03, *p* = 0.68	rho = 0.06, *p* = 0.40	rho = −0.18, *p* = 0.17
	1st postop day	rho = −0.02, *p* = 0.77	rho = 0.00, *p* = 0.97	rho = −0.10, *p* = 0.44
	4th postop day	rho = 0.09, *p* = 0.10	rho = 0.13, *p* = 0.10	rho = −0.04, *p* = 0.77

**Table 3:** Blood cell count indexes and hospital stay in the global cohort and the open and VATS surgery groups.

		Hospital Stay (All)	Hospital Stay (Open)	Hospital Stay (VATS)
**NLR**	Preoperative	rho = 0.12, *p* = 0.06	rho = 0.13, *p* = 0.08	rho = 0.06, *p* = 0.67
	1st postop day	rho = 0.24, *p* = 0.004	rho = 0.16, *p* = 0.043	rho = 0.12, *p* = 0.38
	4th postop day	rho = 0.23, *p* = 0.001	rho = 0.19, *p* = 0.013	rho= 0.10, *p* = 0.51
**PLR**	Preoperative	rho = 0.04, *p* = 0.55	rho = 0.04, *p* = 0.61	rho = 0.16, *p* = 0.22
	1st postop day	rho = 0.11, *p* = 0.10	rho = 0.13, *p* = 0.09	rho = 0.16, *p* = 0.23
	4th postop day	rho = 0.19, *p* = 0.007	rho = 0.17, *p* = 0.03	rho = 0.27, *p* = 0.07
**LMR**	Preoperative	rho = −0.13, *p* = 0.046	rho = −0.11, *p* = 0.13	rho = 0.05, *p* = 0.71
	1st postop day	rho = −0.16, *p* = 0.016	rho = −0.15, *p* = 0.055	rho = 0.02, *p* = 0.88
	4th postop day	rho = −0.11, *p* = 0.10	rho = −0.11, *p* = 0.14	rho = 0.11, *p* = 0.46
**SIRI**	Preoperative	rho = 0.17, *p* = 0.008	rho = 0.20, *p* = 0.009	rho = 0.00, *p* = 0.97
	1st postop day	rho = 0.21, *p* = 0.002	rho = 0.21, *p* = 0.007	rho = −0.02, *p* = 0.87
	4th postop day	rho = 0.17, *p* = 0.014	rho = 0.17, *p* = 0.03	rho = −0.14 *p* = 0.36
**AISI**	Preoperative	rho = 0.18, *p* = 0.005	rho = 0.21, *p* = 0.006	rho = 0.06, *p* = 0.64
	1st postop day	rho = 0.22, *p* = 0.001	rho = 0.24, *p* = 0.002	rho = 0.08, *p* = 0.55
	4th postop day	rho = 0.17, *p* = 0.01	rho = 0.18, *p* = 0.02	rho = −0.02, *p* = 0.88

## Data Availability

The data presented in this study are available on request from the first authors.
